# Severe ARDS due to disseminated toxoplasmosis in a person with HIV successfully treated with ECMO: a case report

**DOI:** 10.3389/fmed.2024.1446762

**Published:** 2025-01-13

**Authors:** Daniel Medina, Renzo Ávila, Graciela Rodríguez-Sevilla, Víctor Gumucio, María Bonilla, Gabriela Abelenda-Alonso, Jordi Niubó, Maria Saumoy, Juan Tiraboschi, María Paz Fuset, Arkaitz Imaz

**Affiliations:** ^1^Human Immunodeficiency Virus (HIV) and Sexually Transmitted Infections (STI) Unit, Department of Infectious Diseases, Bellvitge University Hospital, Bellvitge Biomedical Research Institute (IDIBELL), University of Barcelona, L’Hospitalet de Llobregat, Barcelona, Spain; ^2^Department of Intensive Care Medicine, Bellvitge University Hospital, Bellvitge Biomedical Research Institute (IDIBELL), University of Barcelona, L’Hospitalet de Llobregat, Barcelona, Spain; ^3^Department of Microbiology, Bellvitge University Hospital, Bellvitge Biomedical Research Institute (IDIBELL), University of Barcelona, L’Hospitalet de Llobregat, Barcelona, Spain; ^4^Department of Infectious Diseases, Bellvitge University Hospital, Bellvitge Biomedical Research Institute (IDIBELL), University of Barcelona, L’Hospitalet de Llobregat, Barcelona, Spain

**Keywords:** disseminated toxoplasmosis, HIV, ARDS, ECMO, case report

## Abstract

Disseminated toxoplasmosis (DT) is an uncommon but severe complication associated with acquired immunodeficiency syndrome (AIDS). This case report describes the successful treatment of a 28-year-old male individual with AIDS who developed severe and life-threatening acute respiratory distress syndrome (ARDS) resulting from DT, in which extracorporeal membrane oxygenation (ECMO) was employed effectively.

## Introduction

Toxoplasmosis, caused by the protozoan parasite *Toxoplasma gondii*, is a disease that is found worldwide and is associated with a wide range of clinical manifestations. Although toxoplasmosis is often asymptomatic or causes a mononucleosis-like syndrome in immunocompetent individuals, it can lead to severe complications in immunocompromised hosts, particularly those with acquired immunodeficiency syndrome (AIDS) or who have undergone hematopoietic stem-cell transplantation. While primarily known for causing encephalitis in the form of brain abscesses, toxoplasmosis can affect any organ, with ocular and pulmonary manifestations being the most common extracerebral forms. It can occasionally result in disseminated toxoplasmosis (DT) (1), which can trigger a systemic inflammatory response, leading to complications such as shock, acute respiratory failure, hemophagocytic lymphohistiocytosis (HLH), and cardiac dysfunction, with a mortality rate exceeding 80% (2).

The case report presented here outlines the successful treatment of a 28-year-old man with AIDS who developed severe acute respiratory distress syndrome (ARDS) resulting from DT, in which extracorporeal membrane oxygenation (ECMO) was employed effectively.

## Case report

A 28-year-old man, originally from Cuba and relocated to Spain six months before being admitted to our hospital, was diagnosed with human immunodeficiency virus (HIV) infection after experiencing a 3-week history of fever, diarrhea, and abdominal pain. The route of HIV acquisition was sexual transmission. At the time of diagnosis, his CD4+ T-lymphocyte count was 14 cells/μL (normal range [NR] 450–1,240) and his plasma HIV-1 RNA was 4,222,750 copies/mL. He had no significant past medical, psychiatric, or family history, other than being a former smoker. Before initiating antiretroviral therapy (ART), several tests were conducted to rule out any opportunistic infections. However, before the test results were available, he presented to the emergency room (ER) with a rapid onset of dry cough and somnolence over the previous 4 days.

Upon physical examination, it was noted that he had a fever of 39°C, a heart rate of 120 bpm, and a respiratory rate of 22 rpm, despite an oxygen saturation of 99%. Signs of mucocutaneous dehydration were evident. Abdominal examination revealed 1 cm of hepatomegaly. Lung auscultation and neurological examination were unremarkable. The initial chest X-ray indicated subtle bilateral interstitial infiltrates.

Blood tests revealed elevated acute-phase reactants (C-reactive protein of 94.4 mg/L [NR ≤5.0] and ferritin exceeding 80,000 μg/L [NR 30–400]), anemia (hemoglobin 111 g/L [NR 130–165]), thrombocytopenia (44,000/μL [NR 149,000-303,000]), and leukopenia (3,100/μL [NR 1,300-3,400]), predominantly due to lymphopenia. Furthermore, there were increased levels of alanine aminotransferase, aspartate aminotransferase, and lactate dehydrogenase (230 IU/L [NR ≤40], 664 IU/L [NR ≤39], and 5,721 IU/L [NR ≤224], respectively).

**Figure 1:**
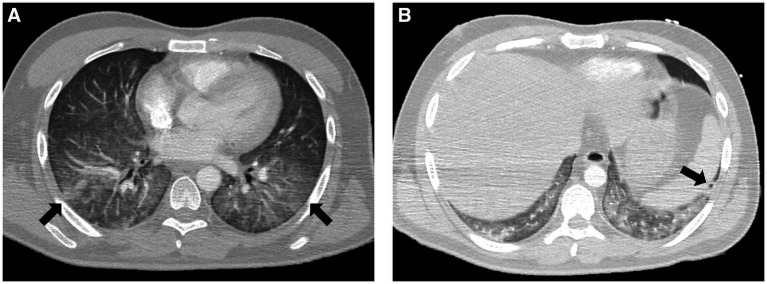
**(A)** Extensive ground-glass opacities in both lung fields (arrows) identified by a chest CT scan. **(B)** Pseudonodular opacity with central cavitation in the left lower lobe (arrow).

His clinical condition deteriorated rapidly upon arrival at the ER, presenting with severe acute respiratory failure requiring orotracheal intubation, invasive mechanical ventilation (IMV), and transfer to the intensive care unit (ICU). An urgent chest computed tomography (CT) scan revealed extensive ground-glass opacities in both lung fields, some with a pseudonodular appearance and one in the left lower lobe with central cavitation (Figures 1A,B).

Empirical treatment was initiated using broad-spectrum antimicrobials, including cotrimoxazole and foscarnet. Due to a high HScore (3) of 286 and a strong suspicion of secondary HLH, methylprednisolone was also administered at a dose of 1 mg/kg/day. Within the initial hours in the ICU, the patient’s respiratory condition deteriorated into ARDS, with an arterial partial pressure of oxygen (PaO2) of 60 mmHg, despite receiving a fraction of inspired oxygen (FiO2) of 100%, resulting in a PaO2/FiO2 ratio of 60 mmHg. Prone positioning and inhaled nitric oxide did not yield a response, prompting the initiation of veno-venous (VV) ECMO via femoro-jugular cannulation (23F and 16F cannulae, respectively) using the Cardiohelp System (Maquet Getinge Cardiopulmonary AG, Rastatt, Germany). The cannulation process was complicated by a jugular vein injury, necessitating a switch to a femoro-femoral VV ECMO configuration, and the damaged vein was surgically repaired.

*Pneumocystis jirovecii* pneumonia (PJP) was ruled out by both antigen detection using immunofluorescence and NAATs performed on deep respiratory samples (bronchoalveolar aspirate and lavage), all of which yielded negative results. Other fungal infections, including disseminated histoplasmosis, were excluded based on negative fungal cultures. Moreover, both tuberculosis and non-tuberculous mycobacterial infections were excluded by mycobacterial cultures and nucleic acid testing (NAATs) for the *Mycobacterium tuberculosis* complex in multiple samples (respiratory tract, peripheral blood, bone marrow, urine, and stool), all of which also returned negative results.

**Figure 2:**
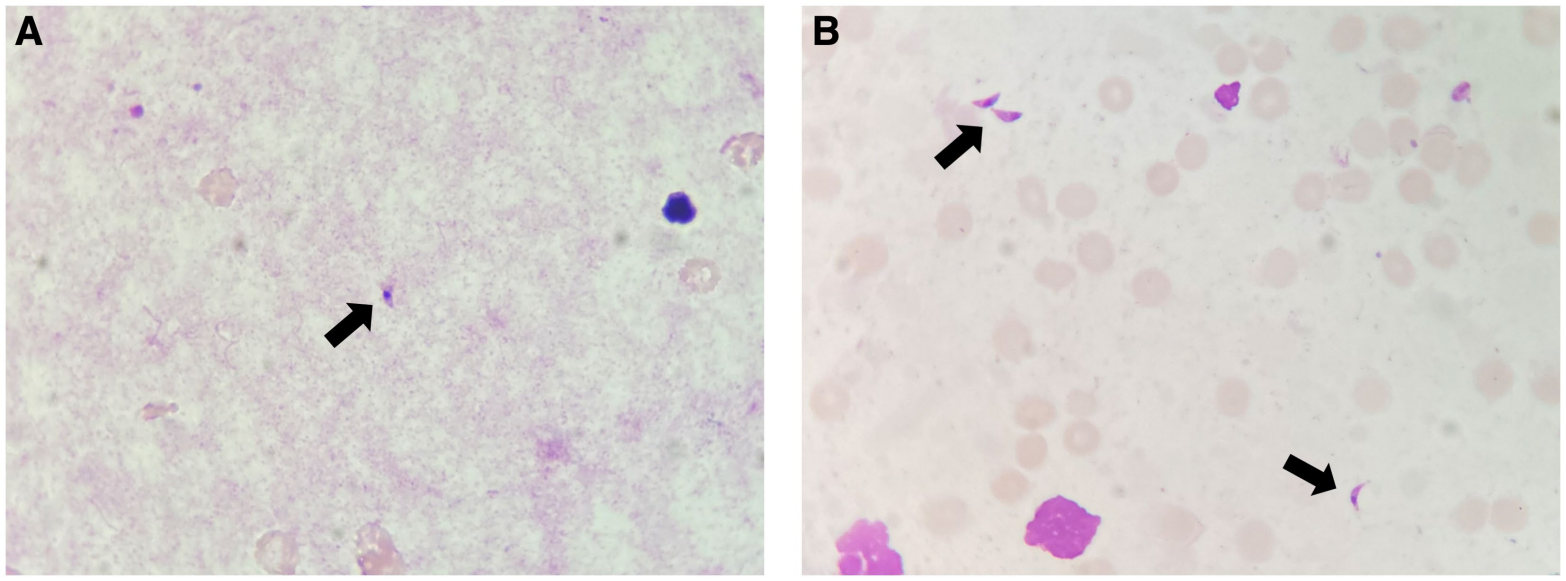
**(A)** *T. gondii* tachyzoites (arrow) identified by Giemsa staining in the bronchoalveolar lavage sample. **(B)** *T. gondii* tachyzoites (arrows) identified by Giemsa staining in the bone marrow aspirate sample.

Further diagnostic tests, including Giemsa staining of the bronchoalveolar lavage (Figure 2A) and bone marrow (Figure 2B) samples, identified parasitic forms consistent with *T. gondii* tachyzoites. NAATs for *T. gondii* were positive in both samples, using an in-house polymerase chain reaction method as previously described (4). The peripheral blood parasite examination was negative. Serum IgG levels for *T. gondii* were 13,000 IU/mL, and IgM was negative (Elecsys Toxo IgG and Elecsys Toxo IgM, Roche Diagnostics, Mannheim, Germany). Blood cytomegalovirus (CMV) viral load was measured at 12,849 IU/mL. A fundoscopic examination revealed bilateral non-specific foci of retinitis, and a non-contrast-enhanced CT scan did not show any intracranial space-occupying lesions (SOLs).

Based on these findings, the patient was diagnosed with DT. Targeted treatment with sulfadiazine, pyrimethamine, and folinate was initiated on day 4, while ART consisting of dolutegravir and emtricitabine/tenofovir alafenamide was started on day 10. Foscarnet was continued due to CMV replication and was subsequently replaced with valganciclovir for secondary prophylaxis. The remaining empiric antimicrobials were gradually discontinued.

Over the following weeks, the patient exhibited a slow but favorable clinical course. ECMO support was discontinued on day 18. Respiratory weaning from IMV to high-flow oxygen therapy was initiated on day 50, and steroid treatment was progressively tapered and eventually discontinued.

**Figure 3:**
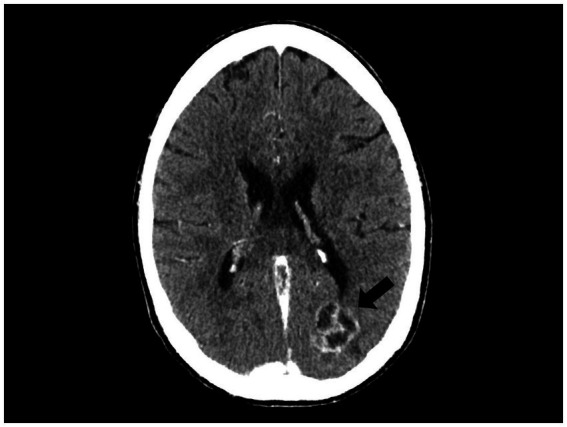
Multicystic lesions with peripheral and septal contrast enhancement (arrow) identified by a brain CT scan, suggestive of toxoplasmic encephalitis.

The onset of agitation with visual hallucinations prompted a contrast-enhanced CT scan on day 71 (Figure 3), which revealed two SOLs in the left occipital lobe and left cerebellar hemisphere, each with a maximum diameter of 29 mm and 19 mm, respectively. Both lesions were multicystic, exhibited peripheral and septal contrast enhancement without perilesional edema, and were suggestive of toxoplasmic encephalitis (TE).

Between weeks 10 and 12, sulfadiazine and pyrimethamine were replaced with clindamycin plus pyrimethamine for 9 days and atovaquone for 5 days due to liver toxicity and severe neutropenia. At week 12, after liver enzyme normalization and neutrophil count recovery, secondary prophylaxis with clindamycin and pyrimethamine and inhaled pentamidine for primary prophylaxis of *Pneumocystis jirovecii* pneumonia (PJP), was initiated.

After a 3-month hospitalization, the patient was discharged. At that time, the CD4+ T-lymphocyte count and plasma HIV-1 RNA load were 260 cells/μL and 658 copies/mL, respectively.

## Discussion

DT is a rare condition in people living with HIV (PLWH) in the ART era. This case of a 28-year-old patient diagnosed with DT in the context of AIDS presents a multifaceted clinical picture, including pulmonary involvement and ARDS, a severe but scarcely reported complication.

Neurologic involvement in DT is found in less than half of the patients (5). Therefore, the initial absence of intracranial SOLs was not remarkable. However, TE was identified after more than 10 weeks of treatment, which is likely related to immune reconstitution inflammatory syndrome (IRIS). While central nervous system immune reconstitution inflammatory syndrome (CNS IRIS) due to tuberculosis and *Cryptococcus spp.* is well documented, IRIS associated with TE is rare, likely due to the delicate balance between the host’s immune response and the parasite’s ability to evade it. This equilibrium is disrupted in individuals with low CD4+ T-lymphocyte counts, leading to uncontrolled protozoan proliferation and abscess formation, which are typically symptomatic or identifiable by brain imaging. In a multicenter study, the median time from ART initiation to the diagnosis of TE-related IRIS was 32 days for unmasking and 51 days for paradoxical IRIS. Interestingly, delaying ART initiation by more than 2–3 weeks did not appear to reduce the incidence of IRIS, and the use of dexamethasone appeared beneficial, especially in cases of paradoxical IRIS (6). In our case, the definition of unmasking or paradoxical CNS IRIS remained uncertain as the initial examinations did not reveal CNS involvement. Moreover, the early use of steroids may have contributed to the late presentation of TE. This raises several questions: Whether an early cerebral MRI could have rapidly identified CNS involvement (suggesting paradoxical IRIS) and whether cerebral MRI should be standardized for patients with DT, even in the absence of neurological deficits or CT findings.

HLH was another important clinical aspect in this case. In PLWH, HLH is typically associated with poor immunovirological control and the coexistence of opportunistic infections. The association between HLH and toxoplasmosis in a person with HIV was first described in 1994. Although few cases have been reported since then (7, 8), a recent retrospective analysis of 38 patients admitted to the ICU because of DT found that over 30% met the criteria for HLH (2). The management of HLH is not well established but should include both the identification and treatment of triggers, as well as the initiation of immunosuppressive therapy (9).

However, the most life-threatening complication was the ARDS associated with pulmonary involvement, which was confirmed by the detection of *T. gondii* tachyzoites in bronchoalveolar lavage samples. Pulmonary toxoplasmosis is an uncommon manifestation that can present as interstitial pneumonitis, necrotizing pneumonia, consolidation, or pleural effusion and can lead to acute respiratory failure (1). Notably, while nearly 90% of patients with DT require IMV within 48 h of ICU admission (2), this case represents, to the best of our knowledge, the first successful use of VV ECMO for ARDS due to DT. The use of ECMO in patients with severe comorbidities remains controversial. Although the Extracorporeal Life Support Organization guidelines do not specify absolute contraindications for ECMO (10), certain protocols have historically limited its application in immunosuppressed individuals, including those with HIV (11). However, growing evidence supports the successful use of ECMO in selected patients with HIV experiencing severe respiratory failure, particularly in cases of PJP-induced ARDS, with a survival rate of nearly 70% (11). Indeed, advancements in ART and sophisticated support therapies have narrowed the gap in ICU survival rates between patients with HIV and those without HIV. Therefore, HIV alone should not prevent access to ECMO when available (12). Acknowledging that our patient’s survival may not have been possible in other healthcare settings around the world underscores the critical need to address healthcare disparities and their implications.

In conclusion, a multidisciplinary approach was crucial in the management of DT, including support with VV ECMO for the associated ARDS. This case emphasizes not only the importance of considering intensive therapies for the treatment of severe AIDS-related conditions in the ART era but also the urgent need to address healthcare inequities on a global scale.

## Patient perspective

The patient does not remember the majority of his hospitalization and took several months to recover functionally. However, since regaining consciousness, he has consistently expressed gratitude for all the interventions carried out by the healthcare personnel.

## Data Availability

The original contributions presented in the study are included in the article/supplementary material, further inquiries can be directed to the corresponding author.
